# Preparation and Identification of a Monoclonal Antibody against the Pseudorabies Virus gE Glycoprotein through a Novel Strategy

**DOI:** 10.3390/vetsci10020133

**Published:** 2023-02-09

**Authors:** Zhenyang Guo, Siyu Zhang, Hu Xu, Wansheng Li, Chao Li, Jing Zhao, Bangjun Gong, Qi Sun, Lirun Xiang, Hongyuan Zhao, Qian Wang, Guohui Zhou, Yandong Tang, Tongqing An, Xuehui Cai, Zhijun Tian, Hongliang Zhang, Jinmei Peng

**Affiliations:** 1 State Key Laboratory of Veterinary Biotechnology, Harbin Veterinary Research Institute, Chinese Academy of Agricultural Sciences, Harbin 150001, China; 2 School of Modern Agriculture and Biotechnology, Ankang University, Ankang 725000, China

**Keywords:** PRV, gE, monoclonal antibody, epitopes

## Abstract

**Simple Summary:**

Porcine pseudorabies is a virulent infectious disease of domestic and wild animals caused by pseudorabies virus. Especially since 2011, the prevalence of mutant strains in China has caused severe economic losses to its breeding industry. As a marker protein, gE glycoprotein can distinguish wild strains from vaccine strains. Therefore, we are committed to obtaining a monoclonal antibody against the gE glycoprotein. We are also studying its characteristics to further explore its application value. In this study, we used a novel immunization and screening strategy to prepare a monoclonal antibody and obtained the monoclonal antibody 1H5 against the gE glycoprotein. An indirect immunofluorescence assay revealed that this monoclonal antibody was specific to both classic and variant strains of pseudorabies virus. Subsequently, we identified the linear epitopes of B cells recognized using the monoclonal antibody. The monoclonal antibody 1H5 bound at ^67^RRAG^70^, which is a novel epitope and is conserved in almost all pseudorabies virus strains. This study provides a new idea for the preparation of monoclonal antibodies.

**Abstract:**

Since 2011, pseudorabies virus (PRV) has recurred in several vaccinated pig farms in China. PRV variants with high virulence were found to be the main cause of the outbreaks. In the face of the PRV epidemic, detection of the wild strain is as important as vaccine immunization, so we hoped to achieve differential diagnosis of PRV by obtaining a monoclonal antibody (mAB) that could be used to identify the wild strain. In this study, we used a novel immunization and screening strategy to prepare an mAB and obtained mAB 1H5 against the gE glycoprotein. An immunofluorescence assay (IFA) revealed that this mAB was specific to both classic and variant strains of PRV. Subsequently, we further identified the linear epitopes of B cells recognized using the mAB. The mAB 1H5 bound at ^67^RRAG^70^, which is a novel epitope and is conserved in almost all PRV strains. These findings provide novel insight into the structure and function of PRV proteins, the analysis of antigenic epitope characteristics, and the establishment of antigen or antibody detection methods.

## 1. Introduction

Porcine pseudorabies (PR), also known as Aujeszky’s disease, is a virulent infectious disease of domestic and wild animals caused by the pseudorabies virus (PRV). PRV has a wide range of hosts, such as domestic animals, including pigs, cattle, sheep, and cats, and wild animals, such as brown bears, wild boars, and foxes [1,2]. However, pigs are the natural host of PRV, which mainly infects pregnant sows and newborn piglets [3]. The clinical manifestations of PRV are abortion, stillbirth, and mummy fetus in pregnant sows. Newborn piglets have neurological symptoms, and the mortality rate can reach 100%, causing substantial economic losses in the pig industry. In some countries in Europe and America, PRV has been nearly eradicated from domestic pigs w the differentiating infected animals from vaccinated animals (DIVA) strategy [4]. Bartha-K61 was introduced in China in the 1970s from Hungary [5], and it has been widely used in Chinese swine farms since then. In late 2011, several pig farms in China in which animals were immunized with the Bartha-K61 vaccine exhibited new outbreaks of PR. The new epidemic strains were found to be mainly PRV variants with enhanced virulence, and Bartha-K61 provided only partial resistance to PRV variant challenge in immunized pigs [6]. Since then, PRV variants have gradually replaced classic strains to become the main strains in China [7,8,9].

Among the 11 envelope glycoproteins of PRV, glycoprotein E (gE) is one of the targets of the humoral immune response [10,11]. Moreover, gE is also the primary virulence factor. This glycoprotein, gE, exists as a heterodimer with glycoprotein I (gI), and together, they are virulent and are necessary for anterograde neuronal transport of virus particles [3]. In addition, gI deletion mutants exhibit attenuated neurotoxicity [12]. Interestingly, deletion of the gE gene does not affect the replication, amplification and immunogenicity of virions, but their virulence is greatly reduced [13]. In different strains of PRV, the mutant gE glycoprotein has an aspartic acid insertion at site 48 and site 496, which is a significant difference from the classical strain and is important as a reference for the molecular epidemiological investigation of the mutant [7]. At present, most of the vaccines developed are genetically deleted vaccines containing gE gene deletion [14,15,16]. Therefore, the gE glycoprotein can be used as a marker protein to distinguish vaccine-immunized pigs from wild virus-infected pigs [17]. In recent years, studies have identified three gE B-cell epitopes, located at aa151-aa155, aa148-aa154 and aa161-aa165 [18,19,20]. Monoclonal antibodies (mABs) against these epitopes are produced by immunizing mice with purified fragments of the extracellular domain of the gE protein expressed in a prokaryotic system, and they provide a reference for the research and development of PRV diagnostic reagents and epidemiological investigation.

In this study, we immunized mice with the whole viral protein of the PRV variant HeN1 as an immunogen and obtained gE-specific mAB 1H5. The mAB 1H5 can react well with classic and variant strains of PRV, which will help in the establishment of antigen detection methods. The mAB 1H5 was bound at ^67^RRAG^70^, which is relatively well conserved in PRV genotype I and genotype II. Moreover, this study is the first to identify the shortest B-cell epitope targeting the gE glycoprotein.

## 2. Materials and Methods

### 2.1. Viruses, Cells, Experimental Animals, and Main Reagents

The PRV HeN1 (10^7.59^ TCID50), PRV SC (10^8.57^ TCID50) and PRV Bartha-K61 (10^7.26^ TCID50) strains (accession numbers for the HeN1 and SC strains: KP098534.1 and KT809429.1, respectively; the Bartha-K61 strain is a gene deletion vaccine strain kept in our laboratory, including gE, gI, US9 and US2 deletion) were stored in our laboratory. Marc-145 cells, 293T cells and SP2/0 myeloma cells were stored in our laboratory. The *E. coli* BL21 (DE3) strain and pGEX-6P-1 vector were provided by our laboratory. Female BALB/c mice (aged 6–8 weeks) were purchased from the Laboratory Animal Center of Harbin Veterinary Research Institute, CAAS, Harbin, China.

### 2.2. Immunization of Mice

All procedures involving animals for this study were approved by and performed in accordance with the guidelines of the Animal Ethics Committee and Laboratory Animal Center of Harbin Veterinary Research Institute, respectively. Marc-145 cells were infected with 100 TCID_50_ PRV HeN1 at 80% confluency. Marc-145 cells with typical cytopathic effects (CPEs) were collected at 48 h postinfection (hpi) and subjected to two freeze–thaw cycles; the toxins were collected, and the virions were inactivated with β-propanolactone (1:50,000; Wako, Japan) at 4 °C for 12 h. After inactivation treatment, the toxins containing the inactivator were placed in a 37 °C water bath for 30 min to inactivate the inactivator. The virus solution was centrifuged at 5000 rpm for 40 min at 4 °C, and the supernatant was collected and then centrifuged at 30,000 rpm for 2 h at 4 °C. After centrifugation, the supernatant was discarded, and the precipitate was dissolved and resuspended in phosphate-buffered saline (PBS). After resuspension in PBS, the precipitate was centrifuged again at 1000 rpm for 3 min. The supernatant was collected, and the protein content in the supernatant was measured using spectrophotometry. The sample was then stored at −80 °C until use. Female BALB/c mice (aged 6–8 weeks) were immunized with 100 μg of the whole virus protein prepared above. The first immunization was performed with the same volume of Freund’s complete adjuvant (Sigma, St. Louis, MO, USA), and the second and third immunizations were performed with the same volume of Freund’s incomplete adjuvant (Sigma, St. Louis, MO, USA). After the last immunization, the serum antibody titers of the mice were determined using indirect Enzyme-linked immunosorbent assay (ELISA). When the serum antibody titer reached more than 1:10,000 in the indirect ELISA test, the immunization attempt was considered successful, and the conditions of cell fusion were achieved.

### 2.3. Preparation of the mAB

Three days after the final booster injection, mice were euthanized using carbon dioxide, and then spleen cells were fused with SP2/0 cells using 50% (*v/v*) polyethylene glycol (Sigma, St. Louis, MO, USA). The fused cells were plated in 96-well plates in Dulbecco’s modified Eagle’s medium (DMEM) supplemented with 1% HAT (Sigma, St. Louis, MO, USA) and 20% fetal bovine serum (FBS) (HyClone Laboratories Inc., South Logan, UT, USA). Approximately 10 days after the completion of cell fusion, the cells in the 96-well plate were observed with a microscope, and the cells grew as cell masses at the bottom of the plate. When the size of the cell masses reached approximately 1/4 of the area of each well, positive hybridomas were detected using indirect ELISA and IFA. The positive hybridoma cells were subcloned at least three times using the limited dilution method and maintained in DMEM supplemented with 1% HT (Sigma, St. Louis, MO, USA) and 20% FBS. Every well with cells of the last subclone tested positive using IFA. The purified hybridoma cells were expanded and stored in large quantities. The class and subclass of the mAB were determined using a commercial Mouse Monoclonal Antibody Isotype Elisa Kit (Proteintech) according to the manufacturer’s protocol.

### 2.4. Indirect ELISA

ELISA plates were coated overnight at 4 °C with 100 μL of inactivated PRV HeN1 strain (1 μg/mL) diluted in bicarbonate coating buffer (1.59 g/L Na2CO3 and 2.93 g/L NaHCO3, pH = 9.6). The wells of the plate were washed four times with PBST (PBS containing 0.05% Tween-20) and then blocked with 5.0% skimmed milk in PBST for 2 h at 37 °C. After gradient dilution of mouse serum, 100 μL was added per well, and the plates were incubated for 30 min at 37 °C. The wells were then washed four times with PBST, and 100 μL of horseradish peroxidase (HRP)-conjugated goat anti-mouse IgG (H + L) (Zsbio, Beijing, China) was added at a dilution of 1:5000 for 30 min at 37 °C. Next, plates were washed four times with PBST, and ELISAs were developed using 100 μL of TMB single solution (Life Technologies) for 15 min at 37 °C. The reactions were then terminated with 50 μL/well 2 M H_2_SO_4_, and the absorbance was measured at 450 nm.

### 2.5. IFA

Marc-145 cells were spread in a 96-well cell culture plate (200 μL/well) and infected with 100 TCID_50_ PRV (HeN1, SC, and Bartha K61 strains) at 80% confluency. In addition, 60% of the cells showed CPEs. In addition, 293T cells were cultured in a 96-well cell culture plate to 80% confluency. The 293T cells were transiently transfected with the eukaryotic expression vector pcDNA3.1-HeN1-gE using X-treme-GENE HP DNA Transfection Reagent (Roche, Basel, Switzerland) according to the manufacturer’s protocol. The culture medium for the above two types of cells was discarded. Then, the cells were washed three times with PBS and fixed with precooled absolute ethanol (200 μL/well) for 45 min at 4 °C. Finally, the absolute ethanol in the wells was discarded, and the plate was allowed to dry at room temperature. MAB culture supernatant (100 μL/well) was added to each well and incubated for 45 min at 37 °C. The cells were then incubated with fluorescein isothiocyanate (FITC)-conjugated goat-anti-mouse antibody (Sigma, St. Louis, MO, USA) at a dilution of 1:200 for 30 min at 37 °C and washed three times with PBS. Photographs were taken using an inverted fluorescence microscope (Nikon TS100, Tokyo, Japan).

### 2.6. Western Blotting

The protein samples were mixed with 5 × SDS buffer and boiled in a boiling water bath for 10 min. The gel plate was fixed on the electrophoresis device, 1 × SDS–PAGE electrophoresis buffer was added, and 10–20 μL of sample was added to each well. The electrophoresis voltage was initially set to 80 V and then adjusted to 120 V when the bromophenol blue in the sample had electrophoresed to the bottom of the separating gel. Then, either the gel was stained with Coomassie brilliant blue or the proteins were electrophoretically transferred to a polyvinylidene difluoride (PVDF) membrane. The membrane was blocked for 2 h with 10% skimmed milk in PBST at room temperature and washed three times with PBST. The membrane was then incubated for 2 h at room temperature overnight with GST mAB (1:15,000; Sigma, St. Louis, MO, USA) or supernatant of an mAB as the primary antibody. After rinsing with PBST, the membrane was treated for 1 h at room temperature with IRDye 800 CW goat anti-mouse IgG (H + L) (Li Cor Bio Sciences, Lincoln, NB, USA) as the secondary antibody. Following four washes with PBST, the bound proteins were visualized using an Infrared Imaging System (Odyssey CL × Image Studio).

### 2.7. Preliminary Analysis of Antigenic Epitopes

**Figure 1:**
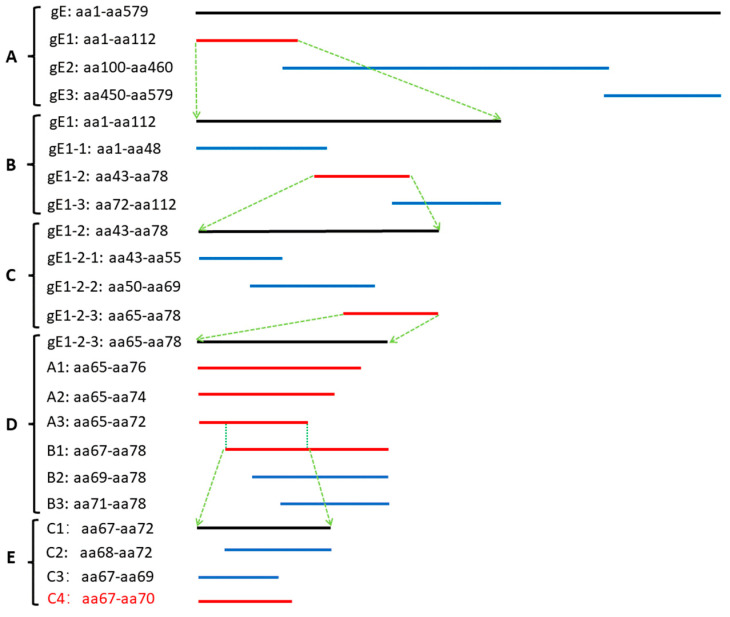
Schematic diagram of the PRV gE glycoprotein showing the expression constructs of the gE glycoprotein fragments. (**A**) The epitopes of mAB 1H5 were studied by expressing gE (aa1-aa579) as three overlapping peptides. (**B**) The epitopes of mAB 1H5 were studied by expressing gE1 (aa1-aa112) as three overlapping peptides. (**C**) The epitopes of mAB 1H5 were studied by expressing gE1-2 (aa43-aa78) as three overlapping peptides. (**D**) The epitopes of mAB 1H5 were studied by expressing gE1-2-3 (aa65-aa78) as six overlapping peptides. (**E**) The epitopes of mAB 1H5 were studied by expressing gE-C1 (aa67-aa72) as three overlapping peptides. The solid red lines in each group represent peptides that react with monoclonal antibodies, while the solid blue lines represent peptides that do not. The two peptides connected by the green dotted lines are the same peptide, and the black line is the enlarged red line.

Three overlapping GST-tagged peptides spanning gE (gE1–gE3; aa1–aa579) were expressed and analyzed using WB to identify the epitopes recognized using mAB 1H5. Then, another three overlapping GST-tagged peptides (gE1-1, gE1-2, gE1-3) were expressed based on the first mapping results. In the same way, another three overlapping GST-tagged peptides (gE1-2-1, gE1-2-1, gE1-2-3) were expressed based on the second mapping results. Then, according to the results of the third localization, amino acids were removed from both ends of the peptide one-by-one (1–2 amino acids were removed each time), and six new GST-tagged peptides (A1, A2, A3, B1, B2, B3) were expressed (Figure 1). Finally, based on the results of the fourth localization, the target peptide segment was truncated to obtain the minimum epitope of the antigen. In addition, we obtained the gE gene sequences of 244 PRV strains from the NCBI database, and Megalign was used to analyze the conformance of epitopes among different PRV strains.

When designing primers, the 5′ ends of the upstream primers and downstream primers were joined with a nucleotide sequence (9–15 bp) that coincided with both ends of the carrier. Homologous recombination reagent (TYHF Biological Science and Technology, China) was used to complete the connection between the target fragment and the vector at 37 °C for 30 min. The homologous recombination products were transformed into *E. coli* BL21 (DE3) cells, which were then inoculated onto Luria-Bertani (LB) plates with ampicillin resistance at 37 °C for 12 h. Ten single colonies from each plate were inoculated in LB medium with ampicillin resistance at 200 rpm and 37 °C for 30 min. Positive clones were selected using PCR and sent to a commercial service for sequencing. Bacterial cultures (100 μL) with correct sequencing results were transferred into 5 mL of LB medium and grown at 170 rpm and 37 °C for 2–3 h, and the OD value of the bacterial culture at 600 nm was determined (0.4–0.6). Then, the bacterial culture (100 μL) was transferred into 5 mL of LB medium again, and 5 μL of isopropyl β-D-1-thiogalactopyranoside (IPTG) was added, after which the cells were grown at 170 rpm and 37 °C for 5 h. In addition, two control groups were set during this process: one contained the empty vector pGEX-6P-1 after induction, and the other group contained the empty vector pGEX-6P-1 without inducer. Finally, recombinant protein expression was detected using western blotting (WB). Next, the truncated recombinant proteins were analyzed using WB, and the truncated recombinant proteins that reacted with the specific mAB were further truncated according to the analysis results. Finally, the shortest epitope recognized by the antibody was determined.

## 3. Results

### 3.1. Immunization of Mice

**Figure 2:**
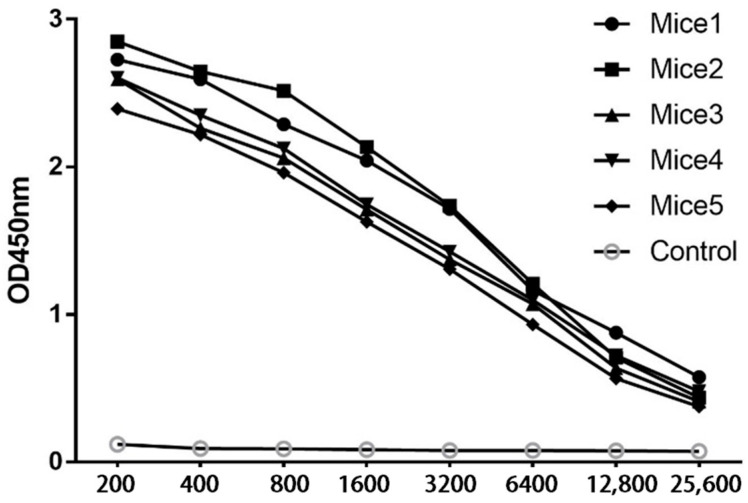
Serum antibody titers of mice were detected using indirect ELISA. Serum was extracted from mice after the third immunization and diluted consecutively, and an indirect ELISA test was performed. The control group was the negative serum of mice treated with the same treatment.

Indirect ELISA was used to measure the serum antibody titer of mice after three immunizations to evaluate the immune effect. The results showed that the response of the mice to the immunogens was good after three immunizations, and the antibody titers were all in the range of 1:10,000–1:12,000 (Figure 2). These results indicated that all the mice met the requirements for fusion.

### 3.2. Preparation of mAB

**Figure 3:**
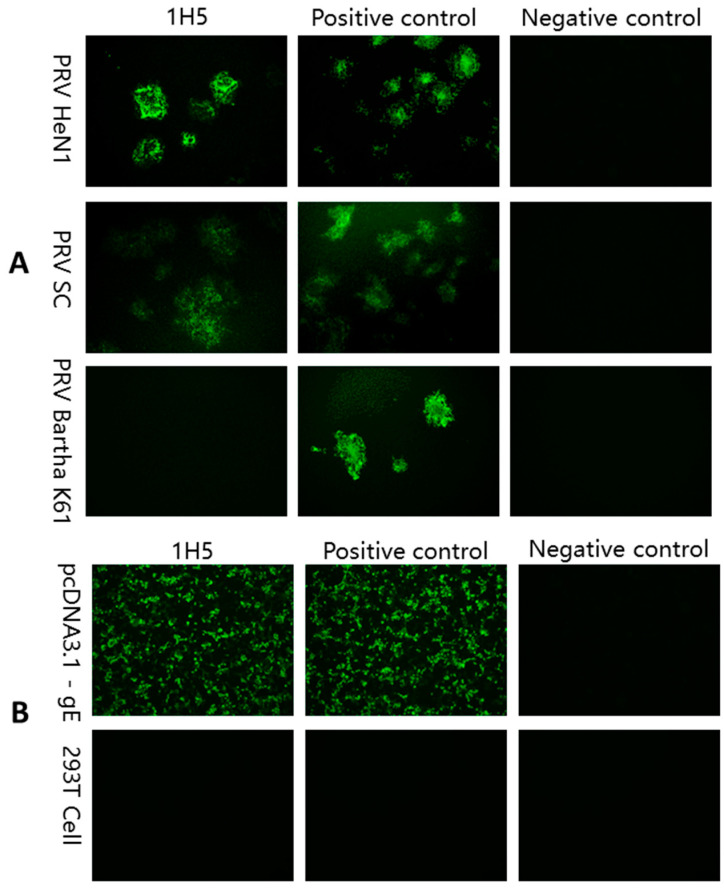
The characteristics of mAB 1H5 were identified using IFA. (**A**) Marc cells infected with PRV (HeN1, SC, Bartha K61) were reacted with the mAB 1H5 supernatant. The positive control was the anti-gB mAB supernatant, and the negative control was the SP20 cell supernatant. (**B**) The recombinant plasmid pcDNA3.1-gE was transfected into 293T cells, and then IFA was performed. The positive control was the anti-gE mAB supernatant, and the negative control was the SP20 cell supernatant.

Splenocytes were obtained from mice with the highest immunogenic antibody titers and fused with SP2/0 myeloma cells. In the first round of screening, Marc-145 cells inoculated with PRV HeN1 or PRV SC strains were used for IFA, and then the positive hybridoma cells that reacted with them were selected for subcloning and naming. In the second round of screening, Marc cells inoculated with the PRV Bartha K61 strain were used for IFA, and then the cell lines that did not react with it were selected. By this screening, an mAB was identified for the protein encoded by the strain with five deleted genes (gE, gI, US9 and US2). Finally, the recombinant plasmid pcDNA3.1-gE was transfected into 293T cells, and the mAB against the PRV gE protein was identified using IFA. Through the above new screening pathway, we obtained an mAB against gE and named it 1H5 (Figure 3). Using a commercially available isotype classification kit, the isotyping results indicated that 1H5 was of the IgG1 subclass. A neutralization assay was performed as described previously [21] and indicated that the mAB did not neutralize PRV (data not shown).

### 3.3. Preliminary Analysis of Antigenic Epitopes

**Figure 4:**
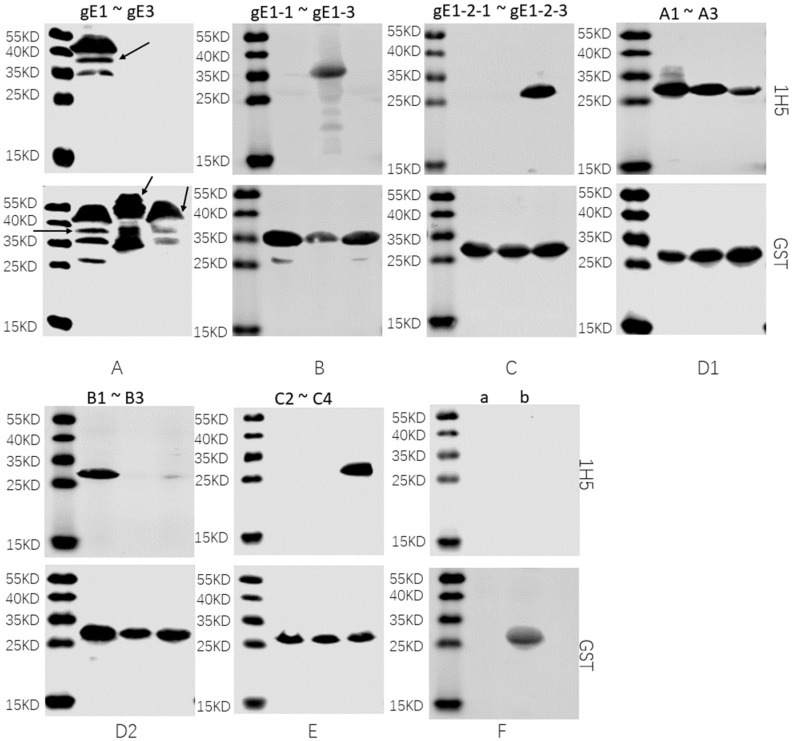
Identification of the linear epitopes recognized using mAB. The truncated gE protein was identified by WB using anti-GST mAB and mAB 1H5 antibodies. (**A**) Three overlapping recombinant peptide fragments, gE1—gE3, spanning aa1-aa579 of gE, were expressed and subjected to WB with mAB 1H5. (**B**) Three overlapping recombinant peptide fragments, gE1-1—gE1-3, spanning aa1-aa112 of gE, were expressed and subjected to WB with mAB 1H5. (**C**) Three overlapping recombinant peptide fragments, gE1-2-1—gE1-2-3, spanning aa43-aa78 of gE, were expressed and subjected to WB with mAB 1H5. (**D**) Six overlapping recombinant peptide fragments, A1—A3 (**D1**) and B1—B3 (**D2**), spanning aa65-aa78 of gE, were expressed and subjected to WB with mAB 1H5. (**E**) Three overlapping recombinant peptide fragments, C2—C3, spanning aa67-aa72 of gE, were expressed and subjected to WB with mAB 1H5. (**F**) WB was performed on the empty PGEX-6P-1 vector and mAB 1H5 before (**a**) and after (**b**) induction of expression.

**Figure 5:**
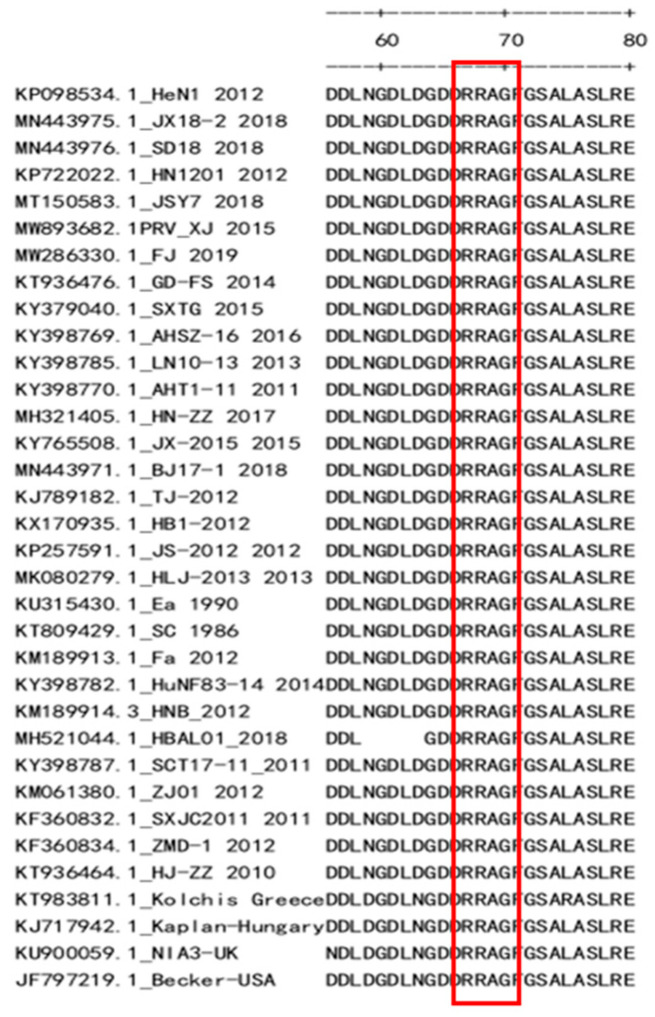
Sequence alignment analysis of the identified epitope. Amino acid sequence analysis of the gE epitopes among the different PRV isolates, the red box shows the amino acid sequence of gE protein at positions 67–70.

To identify the antigenic determinants recognized using mAB 1H5, five rounds of truncated gE and overlapping GST-tagged peptides were subjected to WB. In the first round, three overlapping GST-fused and truncated constructs spanning aa1-aa579 of the HeN1 gE protein were expressed for detection using WB. MAB 1H5 only recognized the fragment located at aa1-aa112 of gE (Figure 4A, the complete western blot figures in the supplementary materials Figure S1). In the second round, mAB 1H5 only recognized the fragment located between aa43 and aa78 of gE (Figure 4B). In the third round, mAB 1H5 only recognized the fragment located at aa65-aa78 of gE (Figure 4C). In the fourth round, the shortest fragment that mAB 1H5 could recognize was the overlapping region between A3 and B1 (aa67–aa72) (Figure 4(D1,D2)). Finally, we further removed the remaining six amino acids, and the results showed that the minimum epitope of the gE protein of PRV recognized using mAB 1H5 was ^67^RRAG^70^ (Figure 4E), and the induced PGEX-6P-1 empty carrier (control group) did not react with 1H5 (Figure 4F). The epitope was conserved in 244 different PRV strains (Figure 5; some representative strains were selected and are shown).

## 4. Discussion

Since 2011, the prevalence of PRV variants in China has caused substantial economic losses in the breeding industry [22,23]. Despite vaccination, epidemiological investigation reports of PRV in China in recent years show that the positive detection rate of PRV in some areas is still 6.9–10.33%, and PRV variants are still the main strains in China [8,9]. Therefore, it is particularly important to develop corresponding antigen or antibody detection methods for epidemic mutant strains. As one of the most important virulence and immunogenic proteins in PRV [24,25], gE was deleted during vaccine development, which laid the foundation for the differential diagnosis of PRV [26].

The selection of antigens is crucial in the preparation of mABs. The prokaryotic system allows large quantities of recombinant proteins to be obtained in a short period of time. Moreover, bacterial cell culture is simple and inexpensive, and the transcription and translation mechanisms in bacteria are well known [27]. However, unlike eukaryotic systems, prokaryotic expression systems often produce proteins that do not fold properly or are inactive or produce endotoxins and proteins not amenable to posttranslational modification [28]. The viral proteins expressed by eukaryotic cells showed complete biological activity, but their expression level was too low [20,29]. In contrast, whole viral proteins not only exhibit complete biological characteristics but are also easy to prepare and purify in large quantities. Although there are some problems with using whole viral proteins as antigens, for example, they cannot be used to selectively prepare a single-protein mAB, it is possible to obtain more types of mABs in this manner. In addition, in this study, we also adopted a new screening strategy to screen fused hybridoma cells; that is, the PRV wild-type strain (HeN1 or SC) and vaccine strain (Bartha K61) were used to infect Marc-145 cells, while 293T cells transfected with the PRV gE plasmid were used for IFA. In combination with this screening strategy, it is possible to obtain a specific mAB by immunization with the whole viral protein. The results showed that our immunization and screening strategies were feasible in the preparation of mABs.

The article mentioned above addresses the preparation of mABs against PRV gE glycoprotein in recent years [18,19,20]. Our study differs from these studies in two main aspects: one is the selection of immunogens, and the other is the screening process. In terms of immunogen selection, it is preferable to use the prokaryotic expression system to express the main antigen-encoding region of gE glycoprotein, which is highly targeted and easy to screen in the next step in the application process. However, we used whole viral proteins from inactivated PRV. The advantage of whole viral proteins is that they retain their natural structure; moreover, they are convenient to prepare in large quantities. In terms of screening methods, they used indirect ELISA and IFA to screen positive hybridoma cells. However, we mainly used IFA to screen for positive hybridoma cells; that is, IFA was performed on Marc cells infected with the PRV wild-type strain and vaccine strain (containing gE gene deletion) and 293T cells transfected with the PRV gE plasmid, which enabled the screening of mAB against the gE glycoprotein. This multiple screening mode provides almost no false positive results, and the operation is convenient. In conclusion, there are significant differences between this study and previous studies in terms of the preparation strategy of mAB. At the same time, according to the experimental results, our new strategy is completely feasible in the preparation of mAB.

**Figure 6:**
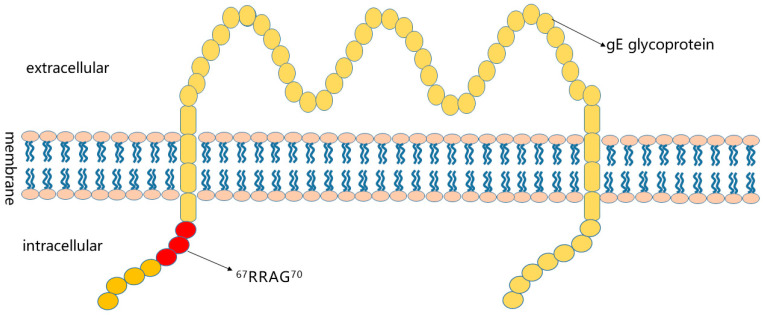
Schematic diagram of the gE glycoprotein transmembrane regions.

In this study, we immunized mice with the whole viral protein of the PRV variant HeN1 as an immunogen and obtained gE-specific mAB 1H5, which reacted well with the PRV HeN1 and PRV SC strains, as shown by IFA. We also conducted a preliminary indirect ELISA on 1H5 and found that it can react with PRV whole virus protein. Further investigation revealed that the epitope of 1H5 was located in the amino acid sequence ^67^RRAG^70^. This is a newly identified epitope, and it is conserved in different PRV genotypes and subtypes. Unfortunately, mAB 1H5 cannot be blocked using positive porcine serum and does not neutralize PRV. There may be differences in the antigenicity of PRV during the infection of different species; therefore, we speculate that mAB 1H5 has a target epitope (^67^RRAG^70^) only in mice and not in pigs. By analyzing studies on the preparation of mABs against gE, we found that prokaryotic expression is more commonly used to obtain antigens [18,19,20]; however, there are few experimental data on the neutralizing activity and blocking efficiency of the mABs in these studies. This may also be related to the inability of prokaryotically expressed proteins to exhibit complete biological activity [28]. In addition, we used correlation analysis software (http://www.Sacs.ucsf.edu/, accessed on 3 December 2022.) [30,31] to reanalyze the structure of the gE glycoprotein and finally concluded that the polypeptide chain passed through the membrane twice: aa1-aa102 and aa455-aa579 are intracellular regions, aa131-aa429 is an extracellular region, and aa103-aa130 and aa430-aa454 are transmembrane regions (Figure 6). Previous researchers have identified the gE glycoprotein as a type I transmembrane protein, in which a polypeptide chain traverses the membrane only once [32]. According to our analysis results, the epitope corresponding to mAb1H5 should be located in the intracellular region of the gE protein. As a result, mABs cannot bind to intact virions. This may also be why mAB 1H5 does not have neutralizing activity. Comparison with the results of previous studies allowed us to show that the mAB prepared by us has completely new epitopes. These findings will help us to further study the structure of the gE glycoprotein and characterize its function. In addition, mAB 1H5 also exhibited good reactivity with classic and variant strains of PRV. This indicates that mAB 1H5 has potential applications in the development of antigen detection methods. In the future, we will continue to explore the ability of mAB 1H5 to capture antigens and develop some antigen detection methods, such as methods using colloidal gold.

## 5. Conclusions

In this study, a novel immunization and screening strategy was used to obtain gE-specific mAB 1H5 with conserved epitopes, which exhibited a good response to both classic and mutant PRV strains. Although 1H5 does not have neutralizing activity and cannot be blocked using positive serum, this mAB is of great significance for the development of PRV antigen detection methods.

## Data Availability

All data pertaining to the study described in the manuscript are described in the report.

## Supplementary Materials

The following supporting information can be downloaded at: https://www.mdpi.com/article/10.3390/vetsci10020133/s1, Figure S1: the complete western blot figures.
