# Supplementary figures and images for: Preparation and Identification of a Monoclonal Antibody against the Pseudorabies Virus gE Glycoprotein through a Novel Strategy
Source: Vet Sci. 2023 Feb 9;10(2):133. doi: 10.3390/vetsci10020133 (PMC9968200; doi:10.3390/vetsci10020133)

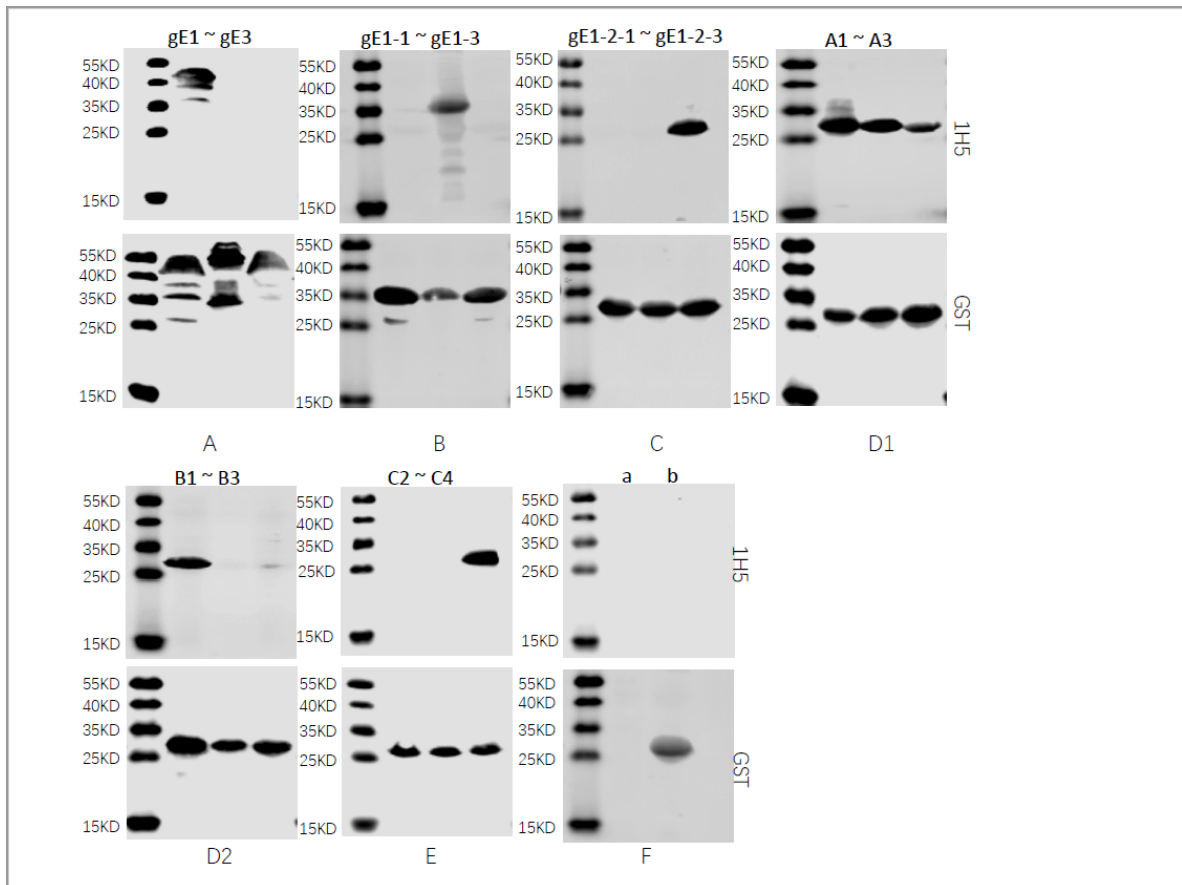

Figure S1: the complete western blot figures.

Supplement: Supplementary file 1 [file vetsci-10-00133-s001.zip › vetsci-2150580-supplementary.pdf]
